# Eosinophilic Chronic Obstructive Pulmonary Disease

**DOI:** 10.1007/s00408-021-00492-0

**Published:** 2021-11-05

**Authors:** Jonathan C. Weissler, Traci N. Adams

**Affiliations:** Division of Pulmonary and Critical Care Medicine, Department of Internal Medicine, University of Texas Southwestern Medical Center, 6201 Harry Hines Blvd, Dallas, TX 75390-9223 USA

**Keywords:** Eosinophilia, COPD, Exacerbation, Interleukin-5, Corticosteroid

## Abstract

Recent therapeutic advances in the management of asthma have underscored the importance of eosinophilia and the role of pro-eosinophilic mediators such as IL-5 in asthma. Given that a subset of patients with COPD may display peripheral eosinophilia similar to what is observed in asthma, a number of recent studies have implied that eosinophilic COPD is a distinct entity. This review will seek to contrast the mechanisms of eosinophilia in asthma and COPD, the implications of eosinophilia for disease outcome, and review current data regarding the utility of peripheral blood eosinophilia in the management of COPD patients.

## Introduction

Eosinophilia has emerged as an important marker for patients with asthma (2) [1, 2], and the presence of eosinophilia in either airway or peripheral blood in asthma patients with difficult to treat disease is an indication for directed therapy [3, 4]. Recently, the presence of eosinophilia in patients with chronic obstructive pulmonary disease (COPD) has drawn increased scrutiny as a possible marker for identifying patients more likely to respond to anti-inflammatory therapies [5, 6] and has led to trials with novel immunologic therapies seeking to target eosinophilia. This review will focus on the mechanisms of eosinophilia in COPD, the utility and specificity of eosinophilia in this patient population, and the data currently available which might validate an approach to “eosinophilic COPD.”

### Mechanisms of Eosinophilia

Eosinophils arise from CD34+ precursor cells, which are the common precursor for both eosinophils and basophils. As opposed to neutrophils, a distinct complex of cytokines and receptors are important for eosinophil maturation. Key to this difference is the expression of a dimeric receptor for interleukin-5 (IL-5). Other than a small population of differentiated basophils, few other immune cells express the receptor for IL-5 [7]. In addition, C–C chemokine receptors, such as RANTES (CCL5) and eotaxin (CCL11), help differentiate the eosinophil and provide a unique pathway for recruitment of eosinophils into peripheral tissues [8]. Following maturation in the bone marrow, eosinophils enter the peripheral circulation under the influence of IL-5. These cells contain a variety of preformed granules capable of up-regulating inflammation and causing injury to lung epithelium. Eosinophils in blood have a circulatory time of 8–18 h [9] before being recruited into a variety of tissues throughout the body including the GI tract (with the exception of the esophagus), the uterus, thymus, and, in some disease states, lung.

The definition of peripheral blood eosinophilia in the literature is elusive, but has become increasingly permissive with the development of anti-cytokine therapies for eosinophilic disorders. Previously, mild eosinophilia was defined as an absolute eosinophil count 500–999 cells/μl, moderate eosinophilia 1000–1499 cells/μl, and severe eosinophilia as > 1500 cells/μl [10]. Normally, the percentage of eosinophils within the peripheral circulation ranges between 1 and 6%. More recently, some authors have lowered the definition of mild eosinophilia to between 351 and 500 cells/μl. In COPD, a variety of definitions have been used in clinical trials, including an eosinophil count greater than 2% of total cells or more than 150 total cells/μl, though this is well within the range of normal variation for eosinophil count. In a large analysis of over 3000 patients with COPD and a history of prior exacerbations, 66% of patients at baseline had an eosinophil count of greater than 2% [11]. Similarly, another study of over 1100 patients with COPD and a history of exacerbations found the median eosinophil count to be 181 cells/μl, with the upper quartile having a median count of 280 eosinophils/μl [12]. A definition of “eosinophilia” in patients with COPD that is actually within accepted normal ranges runs contrary to the experience gained from studies in asthma, where clear demarcation of eosinophilic patients has largely been demonstrated using significantly higher counts [13]. Part of the explanation for the loosening of rigidity for the definition of eosinophilia may relate to the practical aspects of conducting clinical trials for such therapies. The lower the definition of eosinophilia, the more patients who may be enrolled in a trial. Additionally, if benefit is demonstrated, a potentially larger market may exist for a therapy whose initial trials used a lower inclusion value, as witnessed by the varying criteria for use of FDA-approved anti-IL-5 drugs in asthma.

Further complicating the definition and significance of eosinophilia in this patient population is the observation that peripheral blood eosinophil count may vary significantly in the same individual within a 24 h period [9, 14]. In addition, and particularly relevant to COPD, eosinophil counts may be lowered by bacterial infection or concomitant use of corticosteroids, which commonly occur in the setting of an exacerbation [15]. Conversely, 25% of adult patients (mean age 60) who had received parenteral antibiotics but had no clinical evidence of a hypersensitivity reaction had in excess of 500 eosinophils/μl [16], and many medications such as statins and ACE inhibitors may cause asymptomatic eosinophilia in this age group. Of note, COPD patients with low eosinophil counts have less variability than those with higher counts [17].

Cigarette smoking and COPD are largely associated with neutrophilic inflammation and an absolute increase in alveolar macrophages, with lymphocyte responses being less prominent [18]. Multi-cytokine “inflammasomes,” which primarily result in production of Th1 type cytokines such as IL-1*B* and IL-18 and drive neutrophilic inflammation, are increased in lungs of patients with COPD [19]. Recently, interest has also focused on type 2 innate lymphoid cells as inducers of eosinophilia through non-IL-5-dependent mechanisms. Type 2 innate lymphoid cells may be stimulated by a number of cytokines including IL-33, a cytokine which is up-regulated in COPD [20, 21].

Recruitment of inflammatory cells into the lung similarly follows a neutrophil or mononuclear phagocyte emphasis. The CXC chemokines such as CXCL8 are increased in COPD and may increase further during exacerbations [22], resulting in enhanced neutrophil recruitment. However, increased production of RANTES (CCL5) has also been reported in sputum of COPD patients during exacerbations [23].

### Eosinophilia as a Marker of Outcomes in COPD

There are few studies which demonstrate that as a single variable, eosinophilia is associated with poorer outcomes in COPD. Using a “permissive” definition of eosinophilia (> 200 cells/μl or > 2% total cells) in one analysis of 479 patients hospitalized for an initial severe exacerbation of COPD, 36% of patients met the definition of eosinophilia. These patients were shown to have an increased risk of COPD-related readmission and an increased number of emergency department visits within a year of their first admission [24]. Conversely, a study of 243 patients with COPD requiring hospitalization using the same definition of eosinophilia showed that the 25% of patients with eosinophilia on admission had a statistically significant reduction in length of stay as compared to patients without eosinophilia, and no difference in 1 year readmission was observed [25].

A Danish cohort study of 2600 patients with a diagnosis of COPD revealed that an eosinophil count higher than 343 cells/μl had a higher incidence of exacerbations compared to lower levels of eosinophilia in patients followed for 3 years [26]. However, in a Korean study involving longitudinally studied outpatients where the definition of eosinophilia was > 300 cells/μl, no difference in outcome in terms of exacerbation rate was observed, and patients with eosinophilia actually had an improved survival over a 6-year follow-up period [27].

Using a cohort of 2400 current or former smokers, no association was found between peripheral blood eosinophilia (> 200 cells/μl) and exacerbation risk, though some correlation between sputum eosinophilia and overall COPD-related outcomes was observed [28]. A relationship between exacerbation rate and increasing eosinophil counts above the 2% threshold, as compared to those without eosinophilia, could not be demonstrated in 458 patients with COPD and a history of current or past smoking in France [29]. This is in stark contrast to many studies of adult asthma where the presence of significant eosinophilia is associated with markedly worse outcomes including more frequent and more severe exacerbations [30, 31]. Indeed, the association of poorer outcomes in eosinophilic asthma appears particularly strong for patients who are clinically resistant to corticosteroids [10]. Therefore, the relationship between eosinophilia in COPD and response to corticosteroids could be an important clue as to whether a distinct population of COPD patients can be defined by the presence of eosinophilia.

### Eosinophilia as a Marker of Responsiveness to Corticosteroids in COPD

In patients with asthma, different “endotypes” based on clinical presentation, cytokine profile, and the presence of eosinophilia have been proposed as a means toward classifying asthma phenotypes. Significant data have demonstrated the presence of a highly eosinophilic, late-onset asthma group without significant evidence of atopy. This group is highly resistant to conventional doses of inhaled corticosteroids (ICS) [3], with frequent exacerbations and more severe asthma clinically. The mechanism of resistance to corticosteroids in this group is not completely understood but appears to reflect prolonged inflammation, predominantly due to T-helper 2 cytokines, leading to specific alterations in the cellular glucocorticoid receptor (GR) [32, 33], which have not been described in COPD. In patients with COPD, data exist that other mechanisms of glucocorticoid resistance are present, particularly as it pertains to changes in histone deacetylase activity, which leads to a decreased availability of glucocorticoid binding sites in the nucleus [34] and phosphorylation of the GR by serine proteases [35]. The synergistic effect of ICS and long acting inhaled *B*-2 agonists (LABA) may be in part related to an increase in histone deacetylase activity [36]. No data currently link eosinophilia to corticosteroid resistance in COPD and indeed, the data from many studies are quite the opposite.

In patients with a history of exacerbations treated with either an ICS or an ICS/LABA combination, the addition of ICS significantly reduced exacerbation rate [11]. When stratified by baseline eosinophil count, patients with > 2% eosinophils had a more significant response (29% reduction, *p* < 0.001) than those with less than 2% eosinophils, in whom a statistically significant benefit of ICS could not be demonstrated. Moreover, patients with even higher levels of eosinophilia in this study showed a progressively greater benefit of ICS. Other studies have also suggested that patients with low eosinophil counts are poorly responsive to the effects of ICS. In an analysis of the INSPIRE trial comparing an ICS/LABA combination to tiotropium, a long acting anti-muscarinic drug (LAMA), a significant benefit in reducing exacerbations, was found in patients with > 2% eosinophilia at study entry (*p* = 0.006) but not in the < 2% group (*p* = 0.186) [37]. The same analysis found similar outcomes in the TRISTAN study, which compared the ICS/LABA combination to placebo. The group with > 2% eosinophils had a significant reduction in exacerbations following addition of ICS (*p* < 0.001) but those with < 2% eosinophilia did not (*p* = 0.957).

However, the data are not entirely consistent that eosinophilia identifies the subset of COPD patients most likely to benefit from ICS. In one study (ISOLDE), improvement of lung function in patients on ICS was associated with eosinophil counts > 200 cells/μl, although the impact on exacerbation rate was inconsistent, with no difference in time to first moderate/severe exacerbation seen in the eosinophilic group [38]. Indeed, the annual rate of moderate/severe exacerbations was actually lower in patients receiving ICS with less than 200 eosinophils/μl. Furthermore, in the IMPACT trial which analyzed the role of triple therapy (ICS, LABA, LAMA), 43% of patients had eosinophil counts < 150 cells/μl on entry. The annual rate of moderate/severe exacerbations was lower with triple therapy than with a LABA/LAMA combination alone regardless of eosinophil count [39]. In another study (FLAME) which actually demonstrated a superiority of a LAMA/LABA combination to an ICS/LABA combination for reducing exacerbations, there was no difference in responsiveness to the ICS/LABA combination in patients regardless of baseline eosinophil count [40].

Given the conflicting data in individual studies and post hoc analyses of clinical trials, several meta-analyses have been performed to assess the role of ICS in patients with COPD and varying thresholds of eosinophilia. These analyses have demonstrated that ICS does not have a major role in reducing exacerbations in patients with eosinophil counts < 150 cells/μl and relatively increased efficacy in patients with higher eosinophil counts [41, 42]. While there will never likely be a true cutoff defined in the literature, we suggest that eosinophil counts > 300 cells/μl be considered as strongly supportive of ICS initiation along with other factors including exacerbation rate, history of hospitalization for exacerbation, or history of asthma. We also suggest that eosinophil counts < 150 cells/μl be considered as a negative predictor of reduction in exacerbation rate with ICS, along with a history of infrequent exacerbations. While eosinophil counts may vary in an individual patient, lower eosinophil counts tend to be less variable [17], making this a somewhat reliable marker for the absence of response to ICS particularly in patients without a history of frequent exacerbations. It is important to point out that there is currently no published prospective trial which has assessed whether baseline eosinophil count should direct the use of ICS in patients with COPD. All of our current knowledge arises from retrospective analyses of patients with a known history of recent exacerbation who entered into therapeutic trials where ICS were utilized.

There is currently no convincing evidence that eosinophilia correlates with risk of pneumonia in patients on ICS. A recent analysis of 10 trials using ICS in COPD looked for differences in the rate of pneumonia in patients without eosinophilia versus those with > 2% eosinophils. In not a single study did a marginally observed increased risk of pneumonia in patients with low eosinophils on ICS achieve statistical significance, nor did the pooled data show a significant difference in risk of pneumonia while on ICS (*p* = 0.596) [43].

### Anti-IL-5 Therapy for Eosinophilic COPD

Early experience with anti-IL-5 therapies demonstrated that they were effective in dramatically reducing the number of eosinophils in blood and sputum in asthmatics [44, 45], but were ineffective in improving asthma outcomes. This was disappointing given the central role that IL-5 plays in eosinophilia and the clear association between eosinophils and several asthmatic phenotypes. However, it was subsequently appreciated that benefit in patient populations correlated closely with the degree of eosinophilia and several studies have now conclusively validated the role of anti-IL-5 therapy in asthma [13, 46, 47].

There are currently three FDA-approved anti-IL-5 therapies for asthma in the USA. Mepolizumab is a humanized monoclonal IgG1 antibody which directly binds to IL-5 and results in a 78% reduction in blood eosinophils and a 50–55% reduction in bone marrow and lung eosinophils after 4 weeks of therapy. Mepolizumab is indicated for patients with severe eosinophilic asthma and eosinophil counts > 150 at the start of treatment or > 300 within the past 12 months. Clearly, this is a level of eosinophilia which is seen in a substantial number of COPD patients as well.

Benralizumab is a humanized IgG1 antibody directed against the alpha chain of the IL-5 receptor and can block not only the effect of IL-5 but also lead to the depletion of eosinophils in tissue via mechanisms of cellular cytotoxicity [48]. Benralizumab depletes more than 90% of tissue eosinophils and peripheral blood eosinophils within 4 weeks. An eosinophil count of > 300 cells/μl is required within 6 weeks of starting this drug for asthma. The third FDA-approved anti-IL-5 therapy, reslizumab, has not been studied as extensively to date in COPD.

The first large studies using Mepolizumab in COPD were encouraging [49]. Using a patient population with either 150 eosinophils/μl at screening or 300 cells/μl within the preceding 12 months, a lower annual rate of moderate/severe exacerbations was observed with anti-IL-5 therapy compared to placebo. As in studies with asthma, efficacy again tracked with higher eosinophil counts at entry. However, a troubling aspect of the study was that while significant benefit was seen using a lower dose of mepolizumab (100 mg), a significant benefit was not seen using the 300 mg dose, which many clinicians use for higher eosinophil counts. In addition, subsequent FDA analysis maintained that statistical significance for the lower dose was not met using pre-specified criteria. A second trial involving 674 patients submitted to the FDA (MEA117113) failed to demonstrate statistically significant benefit of either the 100 mg or 300 mg dose in reducing moderate/severe exacerbations over a 52 week trial (*p* = 0.068 for low dose, *p* = 0.140 for high dose) compared to placebo [50].

Several studies with benralizumab in eosinophilic COPD have now also been conducted. In the first protocol involving 101 patients randomized to either benralizumab or placebo, no significant reduction in exacerbations was shown [51]. The GALATHEA and TERRANOVA trials evaluated over 2000 patients with COPD in whom benralizumab was added to standard inhaled therapy or COPD over a 56-week period. A statistically significant reduction in moderate/severe exacerbations again was not found [52].

More recently, a Cochrane database analysis of 6 studies with 5542 participants who had COPD and were randomized to anti-IL-5 therapy demonstrated that mepolizumab probably reduced the rate of moderate–severe exacerbations in patients with > 150 eosinophils [53]. In addition, benralizumab reduced the rate of hospitalization in those with an eosinophil count > 220 cells/μl [53]. The promise in pooled data despite failure to show clear benefit in individual studies of anti-IL-5 therapy may suggest that with proper patient selection, a highly specific group of COPD patients might benefit from anti-IL-5 therapy or that the number needed to treat for IL-5 therapies in COPD is quite high such that individual studies had insufficient power to detect a difference. However, the likelihood of anti-IL-5 therapy in COPD being adopted absent clear-cut primary data from a single trial is remote.

### Anti-IL-4/IL-13 Therapy for Eosinophilic COPD

IL-13 is important in the pathophysiology of asthma, as it induces smooth muscle contraction, airway hyper-responsiveness, and goblet cell hyperplasia [54], and along with IL-4, it induces IgE production and generation of C–C cytokines that recruit eosinophils to disease sites. The receptors for IL-4 and IL-13 share the same alpha chain, so while antibodies directed against either cytokine would affect only that cytokine, an antibody directed against the alpha chain of the IL-4 and IL-13 receptor would impact both. Two antibodies have been developed against IL-13 for clinical use, tralokinumab and lebrikizumab. Clinical results with these agents in asthma have been uneven [55]. While tralokinumab has not yet been evaluated in a clinical trial of COPD, a study of lebrikizumab in eosinophilic COPD is underway (NCT02546700).

In contrast to tralokinumab and lebrikizumab, dupilumab, which targets the IL-4/IL-13 receptor alpha subunit, has exhibited significant activity in both eosinophilic and non-eosinophilic asthma. Dupliumab (DPB) is a fully human IgG4 monoclonal antibody directed against the IL-4 R-alpha subunit, and it blocks both IL-4- and IL-13-mediated processes [56]. DPB has resulted in significant improvement in asthma control, airflow, and symptom improvement in patients with asthma poorly controlled by traditional therapy regardless of the presence of baseline peripheral blood eosinophilia. DPB results in an increase in peripheral blood eosinophils while simultaneously improving asthma control by inhibiting the C–C chemokine family and blockade of recruitment into lung [57]. It is FDA approved for the management of severe glucocorticoid-dependent or eosinophilic asthma as well as chronic rhinosinusitis with nasal polyposis and eczema. The BOREAS (a pivotal study to assess the efficacy, safety, and tolerability of dupilumab in patients with moderate-to-severe COPD with type 2 inflammation) study is evaluating the role of DPB in patients with eosinophilic COPD (NCT03930732).

### Anti-IL-33 Therapy for COPD

Although not directly causing eosinophilia, IL-33 is a cytokine produced by “distressed” endothelial and epithelial cells. IL-33 acts as an inducer of Th2 type responses, activates mast cells, and stimulates the release of eosinophil chemotactic proteins and IL-13 production [58]. Recently, a phase 2a trial with Itepekimab, a monoclonal antibody targeting IL-33, was reported in patients with moderate–severe COPD on standard therapy. All patients were current or former smokers aged 40–75. No reduction in exacerbation rate was observed in active smokers, while a significant 42% reduction did occur in former smokers. Overall, the primary endpoint of the trial was not reached [59]. This study may suggest that active smoking, with resultant predominance of neutrophilic inflammation, may cloud the underlying contribution of coexisting TH2 responses to the pathogenesis of COPD.

## Summary

The evidence that eosinophilic COPD constitutes a distinct subset of COPD remains unclear. In contrast to asthma, where the immunologic mechanisms of atopic disease and eosinophilia coalesce with an increasing eosinophil count and poorly controlled asthma, there is relatively little rationale for eosinophilia playing an integral role in the pathogenesis of severe COPD. While eosinophilia in the face of steroid resistance is a hallmark of one common phenotype of severe asthma, data available in COPD actually imply that these patients are more responsive to ICS. The failure of anti-IL-5 therapies, which while successful in reducing eosinophilia had no impact on COPD exacerbation rate in most individual studies, further weakens the concept of eosinophilia as an important contributor to the morbidity of COPD. The positive results reported in meta-analyses may suggest that more precise patient selection could yield different outcomes [53]. In addition, recent studies with IL-33 inhibitors suggest that active smoking may be an important variable in assessing the contribution of TH2 mediators in clinical trials [59].

The utility of peripheral blood eosinophilia as a useful marker in patients with COPD remains uncertain. Absent any data about an increased sensitivity of COPD patients to the effects of eosinophils, the currently accepted definitions of eosinophilia, i.e., > 2% of total cells or > 150 cells/μl, seem arbitrary and of questionable validity as they reside squarely within the accepted normal range. Whether “eosinopenic” COPD truly implies that ICS should not be utilized will be answered only by prospective trials. The specificity of an eosinophil count in a group of patients frequently exposed to corticosteroids and bacteria, which by themselves can lower eosinophil count, may be difficult to ascertain. Given that there are currently 16 million patients with COPD in the USA alone and that Medicare pays $9.65 for a complete blood count with differential, the cost of routinely testing for blood eosinophilia would be in excess of $100 million annually. A robust economic analysis would be necessary to validate this strategy, as alternate therapies for COPD are of comparable cost. In addition, markedly increased health costs in COPD patients receiving ICS, which might be expected given the reported increased risk of pneumonia, have not been observed to date.
